# The FUSION protein crystallization screen

**DOI:** 10.1107/S1600576722001765

**Published:** 2022-03-11

**Authors:** Fabrice Gorrec, Dom Bellini

**Affiliations:** a Structural Studies, MRC Laboratory of Molecular Biology, Francis Crick Avenue, Cambridge, Cambridgeshire CB2 0QH, United Kingdom

**Keywords:** X-ray crystallography, macromolecular crystallization, crystallization screens, protein crystals

## Abstract

The success and speed of atomic structure determination of biological macromolecules by X-ray crystallography depends critically on the availability of diffraction-quality crystals. However, the process of screening crystallization conditions often consumes large amounts of sample and time. An innovative protein crystallization screen formulation called FUSION has been developed to help with the production of useful crystals. The concept behind the formulation of FUSION was to combine the most efficient components from the three MORPHEUS screens into a single screen using a systematic approach. The resulting formulation integrates 96 unique combinations of crystallization additives. Most of these additives are small molecules and ions frequently found in crystal structures of the Protein Data Bank (PDB), where they bind proteins and complexes. The efficiency of FUSION is demonstrated by obtaining high yields of diffraction-quality crystals for seven different test proteins. In the process, two crystal forms not currently in the PDB for the proteins α-amylase and avidin were discovered.

## Abstract 2

The FUSION protein crystallization screen, which integrates 96 unique combinations of additives, is presented and its efficiency is demonstrated.

## 1. Abbreviations

Bicine: *N*,*N*-bis­(2-hy­droxy­ethyl)glycine.

CHAPS: 3-[(3-cholamidopropyl)dimethylammonio]-1-propanesulfonate.

CHAPSO: 3-[(3-cholamidopropyl)dimethylammonio]-2-hydroxy-1-propanesulfonate.

HEPES-Na: 4-(2-hy­droxy­ethyl)­piperazine-1-ethane­sulfonic acid, sodium salt.

LiNaK: MORPHEUS mix of chemicals containing lithium sulfate, sodium sulfate and potassium sulfate.

(MRC) LMB: Medical Research Council Laboratory of Molecular Biology.

MES: 2-(*N*-morpholino)­ethane­sulfonic acid.

MOPS: 3-(*N*-morpholino)­propane­sulfonic acid.

MPD: (*RS*)-2-methyl-2,4-pentane­diol.

NDSB: non-detergent sulfobetaine.

NPS: MORPHEUS mix of chemicals containing sodium nitrate, di-sodium hydrogen phosphate and ammonium sulfate.

PDB: Protein Data Bank (Berman *et al.*, 2000 ▸).

PEG: polyethyl­ene glycol.

PEG MME: polyethyl­ene glycol mono­methyl ether.

TRIS: 2-amino-2-(hy­droxy­methyl)-1,3-propane­diol.

## 2. Introduction

Progress in protein X-ray crystallography during the past decade has been mainly driven by instrument and software developments at synchrotrons, enabling high-throughput data collection and processing. In addition, femtosecond time-resolved serial crystallography with X-ray free-electron lasers (XFELs) now provides access to structural dynamics on femtosecond timescales (Nass *et al.*, 2020 ▸). However, the initial yield of diffraction-quality crystals obtained with novel samples is rate limiting. The underlying problem is that each protein sample has specific characteristics. Notably, each protein has a different surface landscape, while every crystal structure is the result of a number of intermolecular inter­actions that need to occur using the available (although often deformable) surfaces. Most of these interactions are weak, water-mediated or hydro­phobic interactions that can easily lead to irreversible aggregation. In fact, crystallization mostly occurs at high levels of saturation, where most samples tend to aggregate randomly rather than assembling into a highly ordered crystal (Chayen *et al.*, 2010 ▸). Given the large number of parameters controlling protein crystallization, it is not currently possible to predict the right conditions to crystallize a novel sample. Instead, a multitude of variables associated with the experiment must be investigated empirically and often simultaneously (Dessau & Modis, 2011 ▸).

The reagents employed in a given crystallization screen can alter many of these variables. These reagents are categorized into three main groups of crystallization components, namely precipitants to reduce solubility, buffers to control pH and additives. Additives are employed for various reasons, for example to facilitate *de novo* structure determination by experimental phasing (McCoy & Read, 2010 ▸). Most reagents, however, have characteristics that have the potential to alter crystallization in several ways. For example, HEPES-like reagents are not only buffers; they can also alter protein stability, either by affecting the surfactant properties of the solution (Vasconcelos *et al.*, 1996 ▸; Lin & Timasheff, 1996 ▸) or by binding to proteins directly (Newman, 2004 ▸). As a result, a positive impact of a reagent on macromolecular crystallization can rarely be explained rationally, even after the structure has been solved.

A systematic and complete factorial approach to crystallization screen formulation that combines the vast numbers of suitable reagents would contain billions of conditions (Abrahams & Newman, 2021 ▸). However, given the scarcity of most macromolecular samples, the number of conditions trialled must be radically reduced to a realistic level. Therefore, formulation strategies have been designed that cover a relatively large theoretical chemical space while minimizing the number of conditions. A number of screens have been published, and many also commercialized, that are typically made of 96 conditions, a format that is highly amenable to automation. A commonly employed strategy is the ‘incomplete factorial’ sampling of components, where reagents and their combinations are omitted randomly (Carter & Carter, 1979 ▸; Rupp, 2003 ▸) or systematically (Gorrec *et al.*, 2011 ▸). Another popular strategy is termed ‘sparse matrix’, which integrates previously successful crystallization conditions that have been selected empirically against a number of samples (Jancarik & Kim, 1991 ▸), and can be optimized to avoid redundancies (Newman, 2004 ▸; Parker & Newstead, 2012 ▸; Gorrec, 2016 ▸).

An alternative strategy is to add mixes of crystallization additives in each condition to enlarge the sampled chemical space (McPherson & Cudney, 2006 ▸). Newman (2004 ▸) demonstrated that buffer molecules observed as ordered ligands in crystal structures deposited in the PDB could form useful mixes for crystallization screens. Later, an extensive search for small ligands that were found with relatively high numbers of occurrences in the PDB (molecular weight cut-off of 250 Da) was the basis for the original MORPHEUS screen formulation (Gorrec, 2009 ▸). To formulate MORPHEUS, 34 additive reagents were grouped into eight additive mixes according to their chemical nature to increase the coverage of the theoretical chemical space while minimizing cross reactions. In addition, their concentrations were maximized to take into account the expected low-affinity interactions with proteins (Danley, 2006 ▸). Some of the selected PDB-derived ligands were buffers, meaning they could be employed to form three buffer systems. Four highly efficient mixes of precipitants were optimized to provide cryoprotection upon flash freezing of the resulting crystals in liquid nitro­gen, taking advantage of the fact that some precipitants are also cryoprotectants, such as glycerol and ethyl­ene glycol (Tyree *et al.*, 2018 ▸). Finally, the MORPHEUS formulation was simplified by combining the stock solutions of the three main screen components in fixed ratios for all conditions. The resulting sampling of the three main components formed a systematic 3D grid screen with 96 conditions.

Following the success of the original MORPHEUS screen, two derivative screens were designed in a similar way. MORPHEUS II integrated mixes of ligands that were less frequently found as small ligands in the PDB (Gorrec, 2015 ▸), a strategy based on the fact that the corresponding reagents were under-utilized for initial crystallization screening (Gorrec, 2013 ▸). For MORPHEUS III, the main criterion for selecting the crystallization additives was their drug-like properties, rather than their small size and the number of occurrences in the PDB (Gorrec & Zinzalla, 2018 ▸; Sammak *et al.*, 2019 ▸).

Here, we present the latest implementation of the MORPHEUS formulation principles, named FUSION. FUSION essentially combines the most efficient components from the three MORPHEUS screens into a single 96-condition screen. For this, a systematic approach to formulate an incomplete factorial screen was taken. We tested FUSION with seven soluble proteins available commercially and obtained a large number of diffraction-quality crystals, demonstrating its efficiency. X-ray diffraction data collection on the largest crystals from the efficiency testing resulted in 12 unique crystal forms, including unreported crystal forms for the proteins α-amylase and avidin.

## 3. Materials and methods

### 3.1. FUSION sampling of additive mixes

Of the 16 additive mixes from MORPHEUS (Gorrec, 2009 ▸) and MORPHEUS II (Gorrec, 2015 ▸), 14 were integrated into FUSION. To complement the pool of additive mixes, three mixes from the more recent MORPHEUS III screen (Gorrec & Zinzalla, 2018 ▸) and two new additive mixes were incorporated. The two new mixes are

(i) cryo-polyols prepared with the four cryoprotectants already used in MORPHEUS II and an additional polyol reagent (*meso*-erythritol);

(ii) NDSBs made of five non-detergent sulfobetaines (including NDSB 195 and NDSB 256 which were already used in MORPHEUS II).

**Figure 1:**
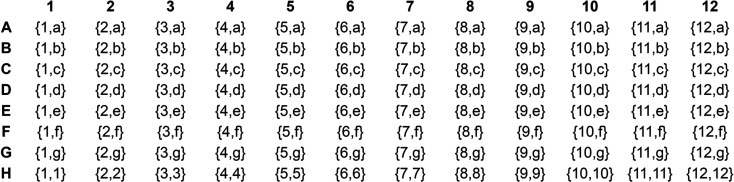
Systematic sampling of the two sets of additive mixes (sets 1 and 2) across the standard 96-well plate layout, forming 96 unique combinations of additives. For example, the combination {1,a} in well A1 corresponds to the two mixes ‘divalent metal cations 1’ (labelled ‘1’ in Table 1 ▸) and ‘alcohols’ (labelled ‘a’ in Table 2 ▸).

As a result, the FUSION sampling of additives is made up of 19 different additive mixes (their preparation requires 81 unique reagents overall). The 19 additive mixes were split into two sets. Set 1 is composed of 12 mixes prepared mostly with reagents that could be categorized as salts (Table 1 ▸). The corresponding PDB ligands are monoatomic or simple polyatomic ions of mineral or organic nature, such as divalent metal cations, halides, carb­oxy­lic acids *etc*. (see Table S1 in the supporting information). Set 2 is mostly composed of small organic compounds with one or more hydroxyl groups as their main functional group (alcohols, polyols, monosaccharides and ethyl­ene glycols) and also surfactants (cholic acid derivatives and NDSBs) (Table 2 ▸). The PDB ligands that gave rise to this other set of additives are listed in Table S2. The two different sets of additive mixes were combined systematically according to a sampling scheme (Fig. 1 ▸).

The 12 mixes forming set 1 were sampled horizontally across the 12 columns of the standard 96-well plate layout and labelled 1–12. The seven mixes from set 2 were sampled vertically across seven of the eight rows (*i.e*. rows A–G) and labelled a–g. The bottom row H is an exception where, instead, the concentration of each mix from set 1 was doubled.

### 3.2. Precipitant mixes and buffer systems

**Figure 2:**
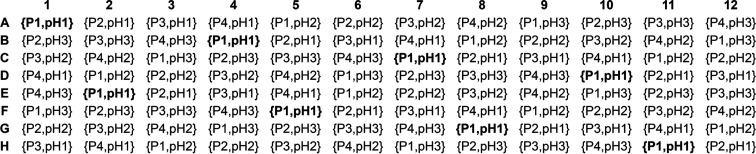
The ‘backbone’ of the FUSION screen, formed with the four precipitant mixes (P1–P4) and three titrated buffer systems (pH1–pH3) from the original MORPHEUS screen. For example, the combination {P1,pH1} in well A1 corresponds to ‘precipitant mix 1’ and ‘buffer system 1’, titrated to pH 6.5 (Table 3 ▸). The combination ‘{P1, pH1}’ is shown in bold to indicate the shift in positions across the 12 columns, in contrast to the original MORPHEUS 3D grid screen, where a single combination of precipitant and buffer populates each column (Gorrec, 2009 ▸). As a result, there are four of each mix of precipitants and three of each buffer system across each row, while there are two of each mix of precipitants and two to three of each buffer system in each column.

Four precipitant mixes and three buffer systems from the original MORPHEUS screen (listed in Table 3 ▸) are used here to form the ‘backbone’ of the FUSION formulation. However, the distribution of these two components is arranged differently across the 96-well plate layout in the FUSION screen, as shown in Fig. 2 ▸.

Before being mixed with the other components, the buffers were titrated to their required pH values (6.5, 7.5 and 8.5) using an IFSET solid-state pH probe (Gorrec, 2009 ▸). Each precipitant mix contains a precipitant that is also a cryoprotectant, namely polyethyl­ene glycol mono­methyl ether 500, ethyl­ene glycol, glycerol or MPD. Their respective final concentrations in the screen (Table 4 ▸) are high enough to vitrify the resulting conditions in a cryo-loop during flash freezing in liquid nitro­gen. Note that the small precipitants/cryoprotectants and the buffers employed here have frequently been reported as ordered ligands in crystal structures deposited in the PDB (Gorrec, 2009 ▸), namely ethyl­ene glycol, glycerol and MPD (PDB residue identifiers EDO, GOL and MPD/MRD, respectively) and the buffers bicine, HEPES, imidazole, MES, MOPS and TRIS (PDB residue identifiers BCN, EPE, IMD, MES, MPO and TRS/TAM, respectively).

### 3.3. Formulation of the FUSION screen

Conditions were prepared by combining the stock solutions according to the following ratios: 0.5 mix of precipitants + 0.1 mix of additives from set 1 + 0.1 mix of additives from set 2 + 0.1 titrated buffer system + 0.2 water. Deviating from this recipe, 15 conditions were prepared with only one additive mix and the ratios became 0.5 mix of precipitants + 0.2 mix of additives from set 1 or 2 + 0.1 titrated buffer system + 0.2 water. This doubles the concentration of a single additive mix in the final solutions in the 12 conditions H1–H12, as described earlier (Fig. 1 ▸). The same applies to the conditions B9, E9 and G9, where the mix ‘oxometallates’ had to be removed as it was causing precipitation.

The incomplete factorial backbone formed with the precipitant mixes and titrated buffer systems (Fig. 2 ▸) was added first, in 96 test tubes, followed by water. The additive mixes from set 1 (Table 1 ▸) were then added to the tubes, and finally the additive mixes from set 2 (Table 2 ▸). Vortex mixing was applied after each addition. The stability of the conditions was assessed regularly over a three-month period by checking the turbidity and pH at 293 and 277 K. The pH of the conditions (IFSET solid-state pH probe) was tested in the tubes at 293 K and also after three months at 277 K. The final pH values of all conditions are listed in Table 4 ▸.

### 3.4. Protein samples

Seven proteins were employed to test the efficiency of the FUSION formulation as an initial screen. All were purchased from Sigma (Table S3). Five proteins were provided as lyophilized powders. After solubilization in water, the lyophilized proteins were left to hydrate for 24 h at 277 K. Insulin and catalase were used as provided (saline/aqueous solutions).

### 3.5. Crystallization experiments

The screen preparation in the test tubes was aliquotted into MRC 96-well crystallization plates (SWISSCI), with the reservoirs containing 85 µl (Gorrec & Löwe, 2018 ▸). Vapour-diffusion sitting-drop experiments (Table 5) were prepared on a Mosquito nanolitre liquid handler (STP Labtech) at 293 K. Equal volumes of protein and reservoir solutions were dispensed (500 nl + 500 nl) with a final mixing step: the ‘advanced transfer options’ on the controlling software were set as follows: four ‘mix cycles’, 400 nl ‘mix volume’, 0.5 mm ‘mix move’. Plates were immediately sealed with three-inch wide Crystal Clear sealing tape (Hampton Research), stored at 291 K and assessed regularly over three weeks using a Leica M205C stereomicroscope.

### 3.6. Crystal screening and structure determination

Crystals were screened for X-ray diffraction with an X-ray diffractometer system comprising an FR-E+ SuperBright rotating anode (Rigaku) and a MAR-DTB image-plate detector. Typically, two images were taken at 0 and 90°, with 1° rotation during 2–20 min exposures. Diffraction patterns were indexed using *iMOSFLM* (Battye *et al.*, 2011 ▸; Powell, 2021 ▸). Diffraction data sets for relatively large crystals (100–500 µm) were collected on beamline I04 (Diamond, Harwell) at 100 K and processed with *DIALS* (Winter *et al.*, 2018 ▸) (Table 6). The resulting structures were solved by molecular replacement with *PHASER* (McCoy *et al.*, 2007 ▸). Interactive atomic model building was performed with *COOT* (Emsley *et al.*, 2010 ▸), refinement with *REFMAC5* (Mushudov *et al.*, 1997 ▸) and *PHENIX* (Liebschner *et al.*, 2019 ▸), and geometric model validation with *MOLPROBITY* (Chen *et al.*, 2010 ▸).

## 4. Results and discussion

### 4.1. Formulation of the FUSION screen

The final formulation of the 96-condition FUSION screen is shown in Table 4 ▸. FUSION can simply be regarded as an evolution of the MORPHEUS formulation and sampling concept. One major difference is the number of additives in FUSION: there are 96 combinations of additives in FUSION, compared with eight in MORPHEUS. Another major difference is that an incomplete factorial approach to formulation was taken because a 3D grid screen against the four precipitant mixes and three buffer systems would have required 96 × 4 × 3 = 1152 conditions. To formulate an incomplete factorial screen with only 96 conditions, the components were sampled systematically, as shown in Figs. 1 ▸ and 2 ▸.

There were practical considerations driving the formulation work. First, although they contain a relatively large number of reagents, the conditions had to be stable, a problem accentuated by the final conditions being fairly saturated. Second, the formulation had to be highly efficient in producing diffracting crystals of a variety of test proteins. Third, the screen had to be practical and cost-efficient. According to this last consideration, the concept of fixed ratios of components was kept, while expensive additive mixes whose usefulness was questionable were excluded.

#### 4.1.1. MORPHEUS additive mixes not integrated into FUSION

The MORPHEUS II mix ‘amino acids II’ was not investigated because it has been cited only once (Wang *et al.*, 2018 ▸) and is expensive to make. Similarly, some mixes from MORPHEUS III, namely ‘dipeptides’, ‘nucleosides’, ‘phytochemicals’ and ‘antibiotics’ were not often requested by our users at the LMB for optimization purposes. Although it is not entirely clear what the underlying reason for this was, they were not investigated to formulate FUSION.

Splitting the additive mixes into two different sets reduced the number of possible cross reactions, but some combinations of mixes were still prone to instability. The ‘lanthanides’ mix from MORPHEUS II, despite producing some exclusive crystal hits (De Munck *et al.*, 2021 ▸; Modenutti *et al.*, 2021 ▸), had to be excluded during preliminary tests because it led to precipitation or flocculation when combined with other mixes.

#### 4.1.2. MORPHEUS additive mixes integrated into FUSION

The counterions from set 1 of additive mixes such as sodium, potassium and others (Table 1 ▸) can alter the physico-chemical properties of proteins in solution (Boström *et al.*, 2011 ▸; Medda *et al.*, 2012 ▸; Salis & Ninham, 2014 ▸; McPherson, 2001 ▸). Additionally, they can facilitate crystallization through the mediation of contact sites, especially with divalent metal cations (Camara-Artigas *et al.*, 2016 ▸; Hegde *et al.*, 2017 ▸). The additives from set 2 (Table 2 ▸) are more likely to alter protein–water interactions (Timasheff, 2002 ▸) than be part of the crystal structure. For example, some polyols and monosaccharides are well known for their stabilizing effects on proteins (Ajito *et al.*, 2018 ▸).

In some cases, crystal structure determination reveals the additives as bound and ordered ligands and enables researchers to understand how the additives help to form or stabilize a crystal structure (De Yoreo & Vekilov, 2003 ▸). For example, the crystal structure of C-phycocyanin was mediated by tetracaine from the MORPHEUS III mix ‘anaesthetic alkaloids’ (PDB code 6yqg, resolution 1.45 Å; Sarrou *et al.*, 2021 ▸). However, this is not a very common scenario, and in many instances the positive impact of additives cannot be explained. For example, the heteromeric complex needed for the initiation of TRIM21 RING-anchored ubiquitin chain elongation crystallized in a MORPHEUS II condition formulated with 1,2,6-hexane­triol as cryoprotectant and the additive mix ‘LiNaK’ (PDB code 7bbd, resolution 2.20 Å; Kiss *et al.*, 2021 ▸), but no extra densities could be attributed to any of these molecules or atoms. In another example, a condition from MORPHEUS II integrating 1,5-pentane­diol as cryoprotectant and the additive mix ‘polyamines’ enabled the crystallization of the complex of RagA/C heterodimer that binds to mTORC1 (PDB codes 6s6a and 6s6d, resolution 2.50–2.63 Å; Anandapadamanaban *et al.*, 2019 ▸). In both cases the electron-density maps did not reveal why the crystallization additives were essential.

#### 4.1.3. Newly formulated additive mixes

Although the PDB ligands in the newly formulated mixes are not especially common in the PDB (Table S2), the two new additive mixes made of cryoprotecting polyols and NDSBs (Table 2 ▸) are potentially very useful. The cryo-polyol additives can tune the effects of the main cryoprotectants found in the precipitant mixes (Table 3 ▸). Cryoprotection parameters such as differential contraction between crystals and mother liquor (Alcorn & Juers, 2010 ▸) and cooling rates on vitrification (Berejnov *et al.*, 2006 ▸) significantly affect the quality of the resulting X-ray diffraction data. Finally, non-detergent sulfobetaines are widely employed to solubilize proteins (Goldberg *et al.*, 1996 ▸), which could be essential to obtain crystals from samples that precipitate readily in crystallization conditions.

### 4.2. Crystallization of test samples and data collection

Table 5 ▸ shows the number of experiments (*i.e.* sitting droplets) counted as crystallization hits during the crystallization efficiency tests with FUSION. Some droplets contained crystals large enough to be mounted directly for data collection. Other droplets were over-nucleated with many small crystals and would be a reasonable basis for optimization experiments (McPherson & Cudney, 2014 ▸; Jones *et al.*, 2019 ▸). Salt crystals occurred rarely during the tests and then always in conditions containing the mixes ‘divalent cations I’ and ‘anaesthetic alkaloids’.

Large single crystals (in duplicates where possible) were harvested from three different FUSION conditions for each test sample. Table 6 ▸ shows the 12 unique crystal forms found out of the 21 non-redundant crystals tested. The diffraction resolutions shown in Table 6 ▸ are comparable to those previously reported in the PDB for the same proteins. Importantly, different crystal forms were obtained for three proteins: catalase, concanavalin A and insulin. We found unreported crystal forms for α-amylase and avidin, which have been deposited in the PDB (PDB codes 7p4w and 7p4z, respectively).

None of the additives from the FUSION conditions could be located confidently in any of the electron-density maps obtained. There are a multitude of possible underlying reasons for this. Perhaps the resolution is too low to show small ordered additives, especially when the binding sites are unknown (Deller & Rupp, 2015 ▸). Or maybe the nature of the intermolecular contacts holding a protein crystal structure is too dynamic (Dimova & Devedjiev, 2018 ▸). In fact, the positive impact of FUSION additives on the yield and variety of crystals obtained was most likely acting elsewhere. Crystallization additives alter the water network surrounding a protein (Qiao *et al.*, 2019 ▸) and can play transient and subtle roles in the early stages of crystal nucleation (Van Driessche *et al.*, 2018 ▸). It should also be considered that additives tune the main experimental parameters such as surface tension, osmolarity and others (Tauchert *et al.*, 2016 ▸). Finally, they can be useful indirectly. For example, detergent-like additives such as cholic acid derivatives (Table 2 ▸) are potentially beneficial to crystal growth by segregating impurities (McPherson *et al.*, 1986 ▸).

## 5. Conclusions

The formulation and preparation of the FUSION screen has been presented. The efficiency of this new screen has been demonstrated by producing hits for test samples in multiple conditions and even crystal forms that had not been reported previously for the corresponding well known proteins. No electron densities for any of the additives from the FUSION conditions were observed in the resulting structures. However, on the basis of our past experience with novel screen formulations and their initial testing, we suggest that FUSION will probably produce useful hits for many other samples.

## 6. Supporting information

The supporting information contains the following additional tables:

Table S1. PDB-derived ligands from set 1 of FUSION mixes of additives (mixes 1–12).

Table S2. PDB-derived ligands from set 2 of FUSION mixes of additives (mixes a–g).

Table S3. Details of the seven commercially available test proteins.

Table S4. Crystallographic data summary table.

## 7. Competing interests

This work was supported by the Medical Research Council, as part of United Kingdom Research and Innovation. FUSION is sold by Molecular Dimensions Ltd (https://www.moleculardimensions.com) as ‘MORPHEUS FUSION’ under an exclusive licence from the MRC and the medical research charity LifeArc (https://www.lifearc.org).

## Supplementary Material

Additional tables. DOI: 10.1107/S1600576722001765/ei5076sup1.pdf

PDB reference: α-amylase, 7p4w

PDB reference: avidin, 7p4z

## Figures and Tables

**Table 1:** Formulation of FUSION set 1 of additive mixes The stock solutions were supplied by Molecular Dimensions Ltd (Sheffield, UK).

Mix name	Label	Chemicals	Catalogue No.	Origin
Divalent metal cations 1	1	0.3 *M* magnesium chloride, 0.3 *M* calcium chloride	MD2-100-70	MORPHEUS I
Divalent metal cations 2	2	5 m*M* manganese chloride, 5 m*M* cobalt chloride, 5 m*M* nickel chloride, 5 m*M* zinc acetate	MD2-100-232	MORPHEUS II
NPS	3	0.3 *M* sodium nitrate, 0.3 *M* disodium hydrogen phosphate, 0.3 *M* ammonium sulfate	MD2-100-72	MORPHEUS I
Carb­oxy­lic acids	4	0.2 *M* sodium formate, 0.2 *M* ammonium acetate, 0.2 *M* tri­sodium citrate, 0.2 *M* sodium potassium L-tartrate, 0.2 *M* sodium oxamate	MD2-100-76	MORPHEUS I
Amino acids	5	0.2 *M* sodium L-glutamate, 0.2 *M* DL-alanine, 0.2 *M* glycine, 0.2 *M* DL-lysine HCl, 0.2 *M* DL-serine	MD2-100-77	MORPHEUS I
LiNaK	6	0.3 *M* lithium sulfate, 0.3 *M* sodium sulfate, 0.3 *M* potassium sulfate	MD2-100-231	MORPHEUS II
Halides	7	0.3 *M* sodium fluoride, 0.3 *M* sodium bromide, 0.3 *M* sodium iodide	MD2-100-71	MORPHEUS I
Alkalis	8	10 m*M* rubidium chloride, 10 m*M* strontium acetate, 10 m*M* caesium acetate, 10 m*M* barium acetate	MD2-100-233	MORPHEUS II
Oxometallates	9	5 m*M* sodium chromate, 5 m*M* sodium molybdate, 5 m*M* sodium tungstate, 5 m*M* sodium orthovanadate	MD2-100-234	MORPHEUS II
Vitamins	10	3% *w*/*v* sodium L-ascorbate, 3% *w*/*v* choline chloride, 3% *v*/*v* D-panthenol, 3% *w*/*v* pyridoxine hydro­chloride, 3% *w*/*v* thi­amine hydro­chloride	MD2-100-314	MORPHEUS III
Polyamines	11	0.1 *M* spermine·4HCl, 0.1 *M* spermidine·3HCl, 0.1 *M* 1,4-di­amino­butane·2HCl, 0.1 *M* DL-ornithine·HCl	MD2-100-238	MORPHEUS II
Anaesthetic alkaloids	12	2% *w*/*v* lidocaine hydro­chloride monohydrate, 2% *w*/*v* procaine hydro­chloride, 2% *w*/*v* proparacaine hydro­chloride, 2% *w*/*v* tetracaine hydro­chloride	MD2-100-320	MORPHEUS III

**Table 2:** Formulation of FUSION set 2 of additive mixes The stock solutions were supplied by Molecular Dimensions Ltd (Sheffield, UK).

Mix name	Label	Chemicals	Catalogue No.	Origin
Alcohols	a	0.2 *M* 1,6-hexane­diol, 0.2 *M* 1-butanol, 0.2 (*RS*)-1,2-propane­diol, 0.2 *M* 2-propanol, 0.2 *M* 1,4-butane­diol, 0.2 *M* 1,3-propane­diol	MD2-100-73	MORPHEUS I
Ethyl­ene glycols	b	0.3 *M* di­ethyl­ene glycol, 0.3 *M* tri­ethyl­ene glycol, 0.3 *M* tetra­ethyl­ene glycol, 0.3 *M* penta­ethyl­ene glycol	MD2-100-74	MORPHEUS I
Monosaccharides 1	c	0.2 *M* D-glucose, 0.2 *M* D-mannose, 0.2 *M* D-galactose, 0.2 *M* L-fucose, 0.2 *M* D-xylose, 0.2 *M* *N*-acetyl-D-glucosamine	MD2-100-75	MORPHEUS I
Monosaccharides 2	d	0.2 *M* xylitol, 0.2 *M* D-(−)-fructose, 0.2 *M* D-sorbitol, 0.2 *M* myo-inositol, 0.2 *M* L-rhamnose monohydrate	MD2-100-236	MORPHEUS II
Cholic acid derivatives	e	3% *w*/*v* CHAPS, 3% *w*/*v* CHAPSO, 3% *w*/*v* sodium glyco­cholate hydrate, 3% *w*/*v* tauro­cholic acid sodium salt hydrate	MD2-100-319	MORPHEUS III
Cryo-polyols	f	5% *w*/*v* 1,2,4-butane­triol, 5% *w*/*v* 1,2,6-hexane­triol, 5% *w*/*v* 1,5 -pentane­diol, 5% *w*/*v* 1,1,1-tris­(hy­droxy­methyl)­propane, 5% *w*/*v* *meso*-erythritol	MD2-100-400	New
NDSBs	g	3% *w*/*v* NDSB 195, 3% *w*/*v* NDSB 201, 3% *w*/*v* NDSB 211, 3% *w*/*v* NDSB 221, 3% *w*/*v* NDSB 256	MD2-100-401	New

**Table 3:** Formulation of stock solutions from the original MORPHEUS screen, containing precipitants and buffers, that were employed to formulate FUSION The labels refer to Fig. 2 ▸, which shows their distribution across the standard 96-well plate layout. The stock solutions were supplied by Molecular Dimensions Ltd (Sheffield, UK).

Mix name	Label	Chemicals	Catalogue No
Precipitant mix 1	P1	20% *w*/*v* PEG 20 000, 40% *v*/*v* PEG MME 500	MD2-250-81
Precipitant mix 2	P2	20% *w*/*v* PEG 8000, 40% *v*/*v* ethyl­ene glycol	MD2-250-82
Precipitant mix 3	P3	20% *w*/*v* PEG 4000, 40% *v*/*v* glycerol	MD2-250-83
Precipitant mix 4	P4	25% *w*/*v* PEG 1000, 25% *w*/*v* PEG 3350, 25% *v*/*v* MPD	MD2-250-84
Buffer system 1	pH1	1 *M* MES, 1 *M* imidazole	MD2-100-100
Buffer system 2	pH2	1 *M* MOPS, 1 *M* HEPES-Na	MD2-100-101
Buffer system 3	pH3	1 *M* bicine, 1 *M* TRIS-base	MD2-100-102

**Table 4:** FUSION formulation Details of the mixes of additives employed to formulate the screen can be found in Tables 1 ▸ and 2 ▸. Conditions can also be found online in the LMB screen database (where FUSION is the plate ‘LMB 23’, https://www2.mrc-lmb.cam.ac.uk/groups/JYL/WWWrobots/robot-nomenclature.html). The ‘Final pH’ column list the pH values as measured experimentally after all components have been combined for each condition.

Well	Mix of precipitants	Buffer system	Mix of additives (set 1)	Mix of additives (set 2)	Final pH
A1	10% *w*/*v* PEG 20 000, 20% *v*/*v* PEG MME 500	0.1 *M* MES/imidazole pH 6.5	30 m*M* of each divalent cation 1	20 m*M* of each alcohol	6.36
A2	10% *w*/*v* PEG 8000, 20% *v*/*v* ethyl­ene glycol	0.1 *M* MES/imidazole pH 6.5	0.5 m*M* of each divalent cation 2	20 m*M* of each alcohol	6.41
A3	10% *w*/*v* PEG 4000, 20% *v*/*v* glycerol	0.1 *M* MES/imidazole pH 6.5	30 m*M* of each NPS	20 m*M* of each alcohol	6.84
A4	12.5% *w*/*v* PEG 1000, 12.5% *w*/*v* PEG 3350, 12.5% *v*/*v* MPD	0.1 *M* MES/imidazole pH 6.5	20 m*M* of each carb­oxy­lic acid	20 m*M* of each alcohol	6.62
A5	10% *w*/*v* PEG 20 000, 20% *v*/*v* PEG MME 500	0.1 *M* MOPS/HEPES-Na pH 7.5	20 m*M* of each amino acid	20 m*M* of each alcohol	6.90
A6	10% *w*/*v* PEG 8000, 20% *v*/*v* ethyl­ene glycol	0.1 *M* MOPS/HEPES-Na pH 7.5	30 m*M* of each LiNaK	20 m*M* of each alcohol	7.37
A7	10% *w*/*v* PEG 4000, 20% *v*/*v* glycerol	0.1 *M* MOPS/HEPES-Na pH 7.5	30 m*M* of each halide	20 m*M* of each alcohol	7.36
A8	12.5% *w*/*v* PEG 1000, 12.5% *w*/*v* PEG 3350, 12.5% *v*/*v* MPD	0.1 *M* MOPS/HEPES-Na pH 7.5	1 m*M* of each alkali	20 m*M* of each alcohol	7.42
A9	10% *w*/*v* PEG 20 000, 20% *v*/*v* PEG MME 500	0.1 *M* bicine/TRIS-base pH 8.5	0.5 m*M* of each oxo­metallate	20 m*M* of each alcohol	8.42
A10	10% *w*/*v* PEG 8000, 20% *v*/*v* ethyl­ene glycol	0.1 *M* bicine/TRIS-base pH 8.5	0.3% of each vitamin	20 m*M* of each alcohol	7.69
A11	10% *w*/*v* PEG 4000, 20% *v*/*v* glycerol	0.1 *M* bicine/TRIS-base pH 8.5	10 m*M* of each polyamine	20 m*M* of each alcohol	8.20
A12	12.5% *w*/*v* PEG 1000, 12.5% *w*/*v* PEG 3350, 12.5% *v*/*v* MPD	0.1 *M* bicine/TRIS-base pH 8.5	0.2% *w*/*v* of each anaesthetic alkaloid	20 m*M* of each alcohol	8.15
B1	10% *w*/*v* PEG 8000, 20% *v*/*v* ethyl­ene glycol	0.1 *M* bicine/TRIS-base pH 8.5	30 m*M* of each divalent cation 1	30 m*M* of each ethyl­ene glycol	7.98
B2	10% *w*/*v* PEG 4000, 20% *v*/*v* glycerol	0.1 *M* bicine/TRIS-base pH 8.5	0.5 m*M* of each divalent cation 2	30 m*M* of each ethyl­ene glycol	8.37
B3	12.5% *w*/*v* PEG 1000, 12.5% *w*/*v* PEG 3350, 12.5% *v*/*v* MPD	0.1 *M* bicine/TRIS-base pH 8.5	30 m*M* of each NPS	30 m*M* of each ethyl­ene glycol	8.50
B4	10% *w*/*v* PEG 20 000, 20% *v*/*v* PEG MME 500	0.1 *M* MES/imidazole pH 6.5	20 m*M* of each carb­oxy­lic acid	30 m*M* of each ethyl­ene glycol	6.56
B5	10% *w*/*v* PEG 8000, 20% *v*/*v* ethyl­ene glycol	0.1 *M* MES/imidazole pH 6.5	20 m*M* of each amino acid	30 m*M* of each ethyl­ene glycol	5.93
B6	10% *w*/*v* PEG 4000, 20% *v*/*v* glycerol	0.1 *M* MES/imidazole pH 6.5	30 m*M* of each LiNaK	30 m*M* of each ethyl­ene glycol	6.48
B7	12.5% *w*/*v* PEG 1000, 12.5% *w*/*v* PEG 3350, 12.5% *v*/*v* MPD	0.1 *M* MES/imidazole pH 6.5	30 m*M* of each halide	30 m*M* of each ethyl­ene glycol	6.37
B8	10% *w*/*v* PEG 20 000, 20% *v*/*v* PEG MME 500	0.1 *M* MOPS/HEPES-Na pH 7.5	1 m*M* of each alkali	30 m*M* of each ethyl­ene glycol	7.34
B9	10% *w*/*v* PEG 8000, 20% *v*/*v* ethyl­ene glycol	0.1 *M* MOPS/HEPES-Na pH 7.5	None	60 m*M* of each ethyl­ene glycol	7.36
B10	10% *w*/*v* PEG 4000, 20% *v*/*v* glycerol	0.1 *M* MOPS/HEPES-Na pH 7.5	0.3% of each vitamin	30 m*M* of each ethyl­ene glycol	6.77
B11	12.5% *w*/*v* PEG 1000, 12.5% *w*/*v* PEG 3350, 12.5% *v*/*v* MPD	0.1 *M* MOPS/HEPES-Na pH 7.5	10 m*M* of each polyamine	30 m*M* of each ethyl­ene glycol	7.22
B12	10% *w*/*v* PEG 20 000, 20% *v*/*v* PEG MME 500	0.1 *M* bicine/TRIS-base pH 8.5	0.2% *w*/*v* of each anaesthetic alkaloid	30 m*M* of each ethyl­ene glycol	8.23
C1	10% *w*/*v* PEG 4000, 20% *v*/*v* glycerol	0.1 *M* MOPS/HEPES-Na pH 7.5	30 m*M* of each divalent cation 1	20 m*M* of each mono­saccharide 1	7.30
C2	12.5% *w*/*v* PEG 1000, 12.5% *w*/*v* PEG 3350, 12.5% *v*/*v* MPD	0.1 *M* MOPS/HEPES-Na pH 7.5	0.5 m*M* of each divalent cation 2	20 m*M* of each mono­saccharide 1	7.40
C3	10% *w*/*v* PEG 20 000, 20% *v*/*v* PEG MME 500	0.1 *M* bicine/TRIS-base pH 8.5	30 m*M* of each NPS	20 m*M* of each mono­saccharide 1	8.39
C4	10% *w*/*v* PEG 8000, 20% *v*/*v* ethyl­ene glycol	0.1 *M* bicine/TRIS-base pH 8.5	20 m*M* of each carb­oxy­lic acid	20 m*M* of each mono­saccharide 1	8.49
C5	10% *w*/*v* PEG 4000, 20% *v*/*v* glycerol	0.1 *M* bicine/TRIS-base pH 8.5	20 m*M* of each amino acid	20 m*M* of each mono­saccharide 1	7.92
C6	12.5% *w*/*v* PEG 1000, 12.5% *w*/*v* PEG 3350, 12.5% *v*/*v* MPD	0.1 *M* bicine/TRIS-base pH 8.5	30 m*M* of each LiNaK	20 m*M* of each mono­saccharide 1	8.49
C7	10% *w*/*v* PEG 20 000, 20% *v*/*v* PEG MME 500	0.1 *M* MES/imidazole pH 6.5	30 m*M* of each halide	20 m*M* of each mono­saccharide 1	6.36
C8	10% *w*/*v* PEG 8000, 20% *v*/*v* ethyl­ene glycol	0.1 *M* MES/imidazole pH 6.5	1 m*M* of each alkali	20 m*M* of each mono­saccharide 1	6.50
C9	10% *w*/*v* PEG 4000, 20% *v*/*v* glycerol	0.1 *M* MES/imidazole pH 6.5	0.5 m*M* of each oxo­metallate	20 m*M* of each mono­saccharide 1	6.65
C10	12.5% *w*/*v* PEG 1000, 12.5% *w*/*v* PEG 3350, 12.5% *v*/*v* MPD	0.1 *M* MES/imidazole pH 6.5	0.3% of each vitamin	20 m*M* of each mono­saccharide 1	5.98
C11	10% *w*/*v* PEG 20 000, 20% *v*/*v* PEG MME 500	0.1 *M* MOPS/HEPES-Na pH 7.5	10 m*M* of each polyamine	20 m*M* of each mono­saccharide 1	7.21
C12	10% *w*/*v* PEG 8000, 20% *v*/*v* ethyl­ene glycol	0.1 *M* MOPS/HEPES-Na pH 7.5	0.2% *w*/*v* of each anaesthetic alkaloid	20 m*M* of each mono­saccharide 1	7.38
D1	12.5% *w*/*v* PEG 1000, 12.5% *w*/*v* PEG 3350, 12.5% *v*/*v* MPD	0.1 *M* MES/imidazole pH 6.5	30 m*M* of each divalent cation 1	20 m*M* of each mono­saccharide 2	6.38
D2	10% *w*/*v* PEG 20 000, 20% *v*/*v* PEG MME 500	0.1 *M* MOPS/HEPES-Na pH 7.5	0.5 m*M* of each divalent cation 2	20 m*M* of each mono­saccharide 2	7.60
D3	10% *w*/*v* PEG 8000, 20% *v*/*v* ethyl­ene glycol	0.1 *M* MOPS/HEPES-Na pH 7.5	30 m*M* of each NPS	20 m*M* of each mono­saccharide 2	7.61
D4	10% *w*/*v* PEG 4000, 20% *v*/*v* glycerol	0.1 *M* MOPS/HEPES-Na pH 7.5	20 m*M* of each carb­oxy­lic acid	20 m*M* of each mono­saccharide 2	7.47
D5	12.5% *w*/*v* PEG 1000, 12.5% *w*/*v* PEG 3350, 12.5% *v*/*v* MPD	0.1 *M* MOPS/HEPES-Na pH 7.5	20 m*M* of each amino acid	20 m*M* of each mono­saccharide 2	6.97
D6	10% *w*/*v* PEG 20 000, 20% *v*/*v* PEG MME 500	0.1 *M* bicine/TRIS-base pH 8.5	30 m*M* of each LiNaK	20 m*M* of each mono­saccharide 2	8.53
D7	10% *w*/*v* PEG 8000, 20% *v*/*v* ethyl­ene glycol	0.1 *M* bicine/TRIS-base pH 8.5	30 m*M* of each halide	20 m*M* of each mono­saccharide 2	8.46
D8	10% *w*/*v* PEG 4000, 20% *v*/*v* glycerol	0.1 *M* bicine/TRIS-base pH 8.5	1 m*M* of each alkali	20 m*M* of each mono­saccharide 2	8.48
D9	12.5% *w*/*v* PEG 1000, 12.5% *w*/*v* PEG 3350, 12.5% *v*/*v* MPD	0.1 *M* bicine/TRIS-base pH 8.5	0.5 m*M* of each oxo­metallate	20 m*M* of each mono­saccharide 2	8.60
D10	10% *w*/*v* PEG 20 000, 20% *v*/*v* PEG MME 500	0.1 *M* MES/imidazole pH 6.5	0.3% of each vitamin	20 m*M* of each mono­saccharide 2	5.91
D11	10% *w*/*v* PEG 8000, 20% *v*/*v* ethyl­ene glycol	0.1 *M* MES/imidazole pH 6.5	10 m*M* of each polyamine	20 m*M* of each mono­saccharide 2	6.40
D12	10% *w*/*v* PEG 4000, 20% *v*/*v* glycerol	0.1 *M* MES/imidazole pH 6.5	0.2% *w*/*v* of each anaesthetic alkaloid	20 m*M* of each mono­saccharide 2	6.47
E1	12.5% *w*/*v* PEG 1000, 12.5% *w*/*v* PEG 3350, 12.5% *v*/*v* MPD	0.1 *M* bicine/TRIS-base pH 8.5	30 m*M* of each divalent cation 1	0.3% *w*/*v* of each cholic acid derivative	8.06
E2	10% *w*/*v* PEG 20 000, 20% *v*/*v* PEG MME 500	0.1 *M* MES/imidazole pH 6.5	0.5 m*M* of each divalent cation 2	0.3% *w*/*v* of each cholic acid derivative	6.51
E3	10% *w*/*v* PEG 8000, 20% *v*/*v* ethyl­ene glycol	0.1 *M* MES/imidazole pH 6.5	30 m*M* of each NPS	0.3% *w*/*v* of each cholic acid derivative	7.05
E4	10% *w*/*v* PEG 4000, 20% *v*/*v* glycerol	0.1 *M* MES/imidazole pH 6.5	20 m*M* of each carb­oxy­lic acid	0.3% *w*/*v* of each cholic acid derivative	6.65
E5	12.5% *w*/*v* PEG 1000, 12.5% *w*/*v* PEG 3350, 12.5% *v*/*v* MPD	0.1 *M* MES/imidazole pH 6.5	20 m*M* of each amino acid	0.3% *w*/*v* of each cholic acid derivative	6.02
E6	10% *w*/*v* PEG 20 000, 20% *v*/*v* PEG MME 500	0.1 *M* MOPS/HEPES-Na pH 7.5	30 m*M* of each LiNaK	0.3% *w*/*v* of each cholic acid derivative	7.32
E7	10% *w*/*v* PEG 8000, 20% *v*/*v* ethyl­ene glycol	0.1 *M* MOPS/HEPES-Na pH 7.5	30 m*M* of each halide	0.3% *w*/*v* of each cholic acid derivative	7.42
E8	10% *w*/*v* PEG 4000, 20% *v*/*v* glycerol	0.1 *M* MOPS/HEPES-Na pH 7.5	1 m*M* of each alkali	0.3% *w*/*v* of each cholic acid derivative	7.60
E9	12.5% *w*/*v* PEG 1000, 12.5% *w*/*v* PEG 3350, 12.5% *v*/*v* MPD	0.1 *M* MOPS/HEPES-Na pH 7.5	None	0.6% *w*/*v* of each cholic acid derivative	7.46
E10	10% *w*/*v* PEG 20 000, 20% *v*/*v* PEG MME 500	0.1 *M* bicine/TRIS-base pH 8.5	0.3% of each vitamin	0.3% *w*/*v* of each cholic acid derivative	7.75
E11	10% *w*/*v* PEG 8000, 20% *v*/*v* ethyl­ene glycol	0.1 *M* bicine/TRIS-base pH 8.5	10 m*M* of each polyamine	0.3% *w*/*v* of each cholic acid derivative	8.23
E12	10% *w*/*v* PEG 4000, 20% *v*/*v* glycerol	0.1 *M* bicine/TRIS-base pH 8.5	0.2% *w*/*v* of each anaesthetic alkaloid	0.3% *w*/*v* of each cholic acid derivative	8.33
F1	10% *w*/*v* PEG 20 000, 20% *v*/*v* PEG MME 500	0.1 *M* bicine/TRIS-base pH 8.5	30 m*M* of each divalent cation 1	0.5% *w*/*v* of each cryo-polyol	7.90
F2	10% *w*/*v* PEG 8000, 20% *v*/*v* ethyl­ene glycol	0.1 *M* bicine/TRIS-base pH 8.5	0.5 m*M* of each divalent cation 2	0.5% *w*/*v* of each cryo-polyol	8.30
F3	10% *w*/*v* PEG 4000, 20% *v*/*v* glycerol	0.1 *M* bicine/TRIS-base pH 8.5	30 m*M* of each NPS	0.5% *w*/*v* of each cryo-polyol	8.56
F4	12.5% *w*/*v* PEG 1000, 12.5% *w*/*v* PEG 3350, 12.5% *v*/*v* MPD	0.1 *M* bicine/TRIS-base pH 8.5	20 m*M* of each carb­oxy­lic acid	0.5% *w*/*v* of each cryo-polyol	8.62
F5	10% *w*/*v* PEG 20 000, 20% *v*/*v* PEG MME 500	0.1 *M* MES/imidazole pH 6.5	20 m*M* of each amino acid	0.5% *w*/*v* of each cryo-polyol	5.95
F6	10% *w*/*v* PEG 8000, 20% *v*/*v* ethyl­ene glycol	0.1 *M* MES/imidazole pH 6.5	30 m*M* of each LiNaK	0.5% *w*/*v* of each cryo-polyol	6.53
F7	10% *w*/*v* PEG 4000, 20% *v*/*v* glycerol	0.1 *M* MES/imidazole pH 6.5	30 m*M* of each halide	0.5% *w*/*v* of each cryo-polyol	6.49
F8	12.5% *w*/*v* PEG 1000, 12.5% *w*/*v* PEG 3350, 12.5% *v*/*v* MPD	0.1 *M* MES/imidazole pH 6.5	1 m*M* of each alkali	0.5% *w*/*v* of each cryo-polyol	6.37
F9	10% *w*/*v* PEG 20 000, 20% *v*/*v* PEG MME 500	0.1 *M* MOPS/HEPES-Na pH 7.5	0.5 m*M* of each oxo­metallate	0.5% *w*/*v* of each cryo-polyol	7.47
F10	10% *w*/*v* PEG 8000, 20% *v*/*v* ethyl­ene glycol	0.1 *M* MOPS/HEPES-Na pH 7.5	0.3% of each vitamin	0.5% *w*/*v* of each cryo-polyol	6.82
F11	10% *w*/*v* PEG 4000, 20% *v*/*v* glycerol	0.1 *M* MOPS/HEPES-Na pH 7.5	10 m*M* of each polyamine	0.5% *w*/*v* of each cryo-polyol	7.26
F12	12.5% *w*/*v* PEG 1000, 12.5% *w*/*v* PEG 3350, 12.5% *v*/*v* MPD	0.1 *M* MOPS/HEPES-Na pH 7.5	0.2% *w*/*v* of each anaesthetic alkaloid	0.5% *w*/*v* of each cryo-polyol	7.18
G1	10% *w*/*v* PEG 8000, 20% *v*/*v* ethyl­ene glycol	0.1 *M* MOPS/HEPES-Na pH 7.5	30 m*M* of each divalent cation 1	0.3% *w*/*v* of each NDSB	7.27
G2	10% *w*/*v* PEG 4000, 20% *v*/*v* glycerol	0.1 *M* MOPS/HEPES-Na pH 7.5	0.5 m*M* of each divalent cation 2	0.3% *w*/*v* of each NDSB	7.46
G3	12.5% *w*/*v* PEG 1000, 12.5% *w*/*v* PEG 3350, 12.5% *v*/*v* MPD	0.1 *M* MOPS/HEPES-Na pH 7.5	30 m*M* of each NPS	0.3% *w*/*v* of each NDSB	7.51
G4	10% *w*/*v* PEG 20 000, 20% *v*/*v* PEG MME 500	0.1 *M* bicine/TRIS-base pH 8.5	20 m*M* of each carb­oxy­lic acid	0.3% *w*/*v* of each NDSB	8.60
G5	10% *w*/*v* PEG 8000, 20% *v*/*v* ethyl­ene glycol	0.1 *M* bicine/TRIS-base pH 8.5	20 m*M* of each amino acid	0.3% *w*/*v* of each NDSB	8.11
G6	10% *w*/*v* PEG 4000, 20% *v*/*v* glycerol	0.1 *M* bicine/TRIS-base pH 8.5	30 m*M* of each LiNaK	0.3% *w*/*v* of each NDSB	8.55
G7	12.5% *w*/*v* PEG 1000, 12.5% *w*/*v* PEG 3350, 12.5% *v*/*v* MPD	0.1 *M* bicine/TRIS-base pH 8.5	30 m*M* of each halide	0.3% *w*/*v* of each NDSB	8.54
G8	10% *w*/*v* PEG 20 000, 20% *v*/*v* PEG MME 500	0.1 *M* MES/imidazole pH 6.5	1 m*M* of each alkali	0.3% *w*/*v* of each NDSB	6.37
G9	10% *w*/*v* PEG 8000, 20% *v*/*v* ethyl­ene glycol	0.1 *M* MES/imidazole pH 6.5	None	0.6% *w*/*v* of each NDSB	6.49
G10	10% *w*/*v* PEG 4000, 20% *v*/*v* glycerol	0.1 *M* MES/imidazole pH 6.5	0.3% of each vitamin	0.3% *w*/*v* of each NDSB	5.89
G11	12.5% *w*/*v* PEG 1000, 12.5% *w*/*v* PEG 3350, 12.5% *v*/*v* MPD	0.1 *M* MES/imidazole pH 6.5	10 m*M* of each polyamine	0.3% *w*/*v* of each NDSB	6.34
G12	10% *w*/*v* PEG 20 000, 20% *v*/*v* PEG MME 500	0.1 *M* MOPS/HEPES-Na pH 7.5	0.2% *w*/*v* of each anaesthetic alkaloid	0.3% *w*/*v* of each NDSB	7.23
H1	10% *w*/*v* PEG 4000, 20% *v*/*v* glycerol	0.1 *M* MES/imidazole pH 6.5	60 m*M* of each divalent cation 1	None	6.30
H2	12.5% *w*/*v* PEG 1000, 12.5% *w*/*v* PEG 3350, 12.5% *v*/*v* MPD	0.1 *M* MES/imidazole pH 6.5	1 m*M* of each divalent cation 2	None	6.31
H3	10% *w*/*v* PEG 20 000, 20% *v*/*v* PEG MME 500	0.1 *M* MOPS/HEPES-Na pH 7.5	60 m*M* of each NPS	None	7.58
H4	10% *w*/*v* PEG 8000, 20% *v*/*v* ethyl­ene glycol	0.1 *M* MOPS/HEPES-Na pH 7.5	40 m*M* of each carb­oxy­lic acid	None	7.51
H5	10% *w*/*v* PEG 4000, 20% *v*/*v* glycerol	0.1 *M* MOPS/HEPES-Na pH 7.5	40 m*M* of each amino acid	None	6.42
H6	12.5% *w*/*v* PEG 1000, 12.5% *w*/*v* PEG 3350, 12.5% *v*/*v* MPD	0.1 *M* MOPS/HEPES-Na pH 7.5	60 m*M* of each LiNaK	None	7.39
H7	10% *w*/*v* PEG 20 000, 20% *v*/*v* PEG MME 500	0.1 *M* bicine/TRIS-base pH 8.5	60 m*M* of each halide	None	8.49
H8	10% *w*/*v* PEG 8000, 20% *v*/*v* ethyl­ene glycol	0.1 *M* bicine/TRIS-base pH 8.5	2 m*M* of each alkali	None	8.45
H9	10% *w*/*v* PEG 4000, 20% *v*/*v* glycerol	0.1 *M* bicine/TRIS-base pH 8.5	1 m*M* of each oxometallate	None	8.57
H10	12.5% *w*/*v* PEG 1000, 12.5% *w*/*v* PEG 3350, 12.5% *v*/*v* MPD	0.1 *M* bicine/TRIS-base pH 8.5	0.6% of each vitamin	None	7.06
H11	10% *w*/*v* PEG 20 000, 20% *v*/*v* PEG MME 500	0.1 *M* MES/imidazole pH 6.5	20 m*M* of each polyamine	None	6.30
H12	10% *w*/*v* PEG 8000, 20% *v*/*v* ethyl­ene glycol	0.1 *M* MES/imidazole pH 6.5	0.4% *w*/*v* of each anaesthetic alkaloid	None	6.37

**Table 5:** Number of hits obtained during the crystallization efficiency testing of FUSION The number of hits results from 672 vapour-diffusion experiments (seven protein samples × 96 FUSION conditions).

Protein	Concentration (mg ml^−1^)	*M*_w_ (kDa)	Hits
α-Amylase	30	56	6
Avidin	20	17	15
Catalase	10	60	23
Concanavalin A	12.5	26.5	31
Insulin	9.5–11.5	5.8	21
Lysozyme	50	14.4	16
Thaumatin	50	22	12

**Table 6:** The 12 unique crystal forms obtained during the efficiency testing of FUSION Crystals were vitrified and assessed by X-ray diffraction. For more details see Table S4.

Protein	Condition (well)	Space group	Unit-cell parameters *a*, *b*, *c* (Å)	*CC*_half_ (high-resolution bin)	Resolution (Å)
α-Amylase	H11	*I*222	65.7, 140.4, 154.2	0.28	2.3
Avidin	H12	*C*2	114.4, 43.3 , 62.3	0.97	2.2
Catalase	C4	*P*2_1_2_1_2_1_ (*A*)	86.4, 140.0, 231.9	0.29	1.9
Catalase	F1	*P*2_1_2_1_2_1_ (*B*)	68.5, 171.9, 192.1	0.31	1.5
Catalase	H4	*P*3_2_21	140.6, 140.6, 101.8	0.34	1.6
Concanavalin A	B2	*P*2_1_2_1_2_1_	66.5, 116.2, 122.7	0.43	1.4
Concanavalin A	D8	*P*2_1_	59.9, 64.2, 126.1	0.33	1.6
Concanavalin A	E8	*I*222	63.9, 88.0, 89.4	0.42	1.6
Insulin	A12	*I*2_1_3	77.7, 77.7, 77.7	0.35	1.6
Insulin	A7	*R*3	79.2, 79.2, 37.3	0.17	1.5
Lysozyme	D4	*P*4_3_2_1_2	78.5, 78.5, 37.0	0.37	1.2
Thaumatin	G8	*P*4_1_2_1_2	57.9, 57.9, 150.8	0.90	1.2
