# Supplementary material for: The FUSION protein crystallization screen
Source: J Appl Crystallogr. 2022 Mar 11;55(Pt 2):310–9. doi: 10.1107/S1600576722001765 (PMC8985600; doi:10.1107/S1600576722001765)
Supplement: Supplementary file 1 [file j-55-00310-sup1.pdf]

JOURNAL OF
APPLIED
CRYSTALLOGRAPHY

Volume 55 (2022)

Supporting information for article:

The FUSION protein crystallization screen

Fabrice Gorrec and Dom Bellini

The FUSION protein crystallisation screen: Supplementary material

Fabrice Gorrec[1] and Dom Bellini

Affiliation

MRC Laboratory of Molecular Biology

Francis Crick Avenue,

Cambridge Biomedical Campus

Cambridge CB2 0QH, UK

[1]Corresponding author

Email: fgorrec@mrc-lmb.cam.ac.uk

Tel: +44 (0) 1223 26 7807

Fax: +44 (0) 1223 26 8305

Contents

Table S1. PDB-derived ligands from the set 1 of FUSION mixes of additives (mixes 1-12).

Table S2. PDB-derived ligands from the set 2 of FUSION mixes of additives (mixes a-g).

Table S3. Details on the 7 commercially-available test proteins.

Table S4. Crystallographic data summary table.

Table S1. PDB-derived ligands from the set 1 of FUSION mixes of additives (mixes 1-12). The corresponding numbers of PDB entries are as of June 2021. Some reagents correspond to two or more PDB-derived ligands (*e.g*., ammonium and sulfate). Some ligands are found in different forms because of different protonation states or isomers, in which case the numbers of the corresponding structures were added (*e.g*., ammonium ion and ammonia). Two compounds, procaine and proparacaine, have not yet been reported as ligands in the PDB. The PDB IDs for the ligands were selected using the RCSB chemical search tool http://ligand-expo.rcsb.org/ld-search.html. The number of occurrences as 'stand-alone PDB ligands' was found using the following URL where the PDB chemical ID must be placed at the end (example for alanine, PDB residue identifier "ALA"): https://www.rcsb.org/ligand/ALA.

Mix name	FUSION sampling	Ligand	Residue ID	No. of PDB structures
Divalents I	1	Calcium ion	CA	12382
Divalents I	1	Magnesium ion	MG	17744
Divalent cations II	2	Cobalt ion	CO	859
Divalent cations II	2	Manganese ion	MN	3824
Divalent cations II	2	Nickel ion	NI	2002
Divalent cations II	2	Zinc ion	ZN	18024
NPS	3	Nitrate ion	NO3	780
NPS	3	Phosphate ions	PO4, PI, 2HP	6393
NPS	3	Sulfate ion	SO4	21724
Carboxylic acids	4	(DL)-Tartaric acid	TAR, TLA	607
Carboxylic acids	4	Acetic acid, acetate ion	ACY, ACT	6784
Carboxylic acids	4	Citric acid, citrate ion	CIT, FLC	1721
Carboxylic acids	4	Formic acid	FMT	1449
Carboxylic acids	4	Oxamic acid	OXM	52
Amino-acids	5	DL-Alanine	ALA, DAL	97
Amino-acids	5	DL-Lysine	LYS, DLY	141
Amino-acids	5	DL-Serine	SER, DSN	102
Amino-acids	5	Glycine	GLY	269
Amino-acids	5	L-Glutamic acid	GLU	325
LiNaK	6	Lithium ion	LI	93
LiNaK	6	Potassium ion	K	3234
LiNaK	6	Sodium ion	NA	9732
Halides	7	Bromide ion	BR	519
Halides	7	Fluoride ion	F	78
Halides	7	Iodide ion	IOD	987
Alkalis	8	Barium ion	BA	200
Alkalis	8	Cesium ion	CS	144
Alkalis	8	Rubidium ion	RB	66
Alkalis	8	Strontium ion	SR	169
Oxometalates	9	Chromate ion	CR	10
Oxometalates	9	Molybdate ion	MOO	38
Oxometalates	9	Orthovanadate ion	VO4	102
Oxometalates	9	Tungstate ion	WO4	56
Vitamins	10	(+)-L-Ascorbate (vitamin C)	ASC	41
Vitamins	10	Choline (vitamin J)	CHT	42
Vitamins	10	Pantothenoic acid (for D-Panthenol)	PAU	14
Vitamins	10	Pyridoxine (water-soluble vitamin B6)	UEG	2
Vitamins	10	Thiamine (vitamin B1)	VIB	12
Polyamines	11	1,4-Diaminobutane	PUT	44
Polyamines	11	DL-Ornithine	ORD, ORN	42
Polyamines	11	Spermidine	SPD	82
Polyamines	11	Spermine	SPM	161
Anaesthetic alkaloids	12	Lidocaine (Xylocaine)	LQZ	2
Anaesthetic alkaloids	12	Procaine	n/a	0
Anaesthetic alkaloids	12	Proparacaine	n/a	0
Anaesthetic alkaloids	12	Tetracaine (Amethocaine)	TE4	6
NPS, Carboxylic acids	3,4	Ammonium ion, ammonia	NH4, NH3	270
multiple	1,2,8,10,12	Chloride ion	CL	14903

Table S2. PDB-derived ligands from the set 2 of FUSION mixes of additives (mixes a-g). Data generated as for Table S1.

Mix name	FUSION sampling	Ligand	Residue ID	No. of PDB structures
Alcohols	a	1-Butanol	1BO	13
Alcohols	a	1,2-(RS)-Propanediol	PGR, PGO	201
Alcohols	a	1,3-Propanediol	PDO	45
Alcohols	a	1,4-Butanediol	BU1	67
Alcohols	a	1,6-Hexanediol	HEZ	133
Alcohols	a	2-Propanol (iso-propanol)	IPA	686
Ethylene glycols	b	Diethylene glycol	PEG	3791
Ethylene glycols	b	Pentaethylene glycol	1PE	837
Ethylene glycols	b	Tetraethylene glycol	PG4	1584
Ethylene glycols	b	Triethylene glycol	PGE	1570
Monosaccharides I	c	D-Galactose	GLA, GAL	236
Monosaccharides I	c	D-Glucose	GLC, BGC	611
Monosaccharides I	c	D-Mannose	MAN, BMA	294
Monosaccharides I	c	D-Xylose (D-Xylitol)	XYL	29
Monosaccharides I	c	L-Fucose	FUC, FUL	143
Monosaccharides I	c	N-Acetyl-D-glucosamine	NAG,NBG	762
Monosaccharides II	d	D-(-)-Fructose	FRU; FUD	59
Monosaccharides II	d	D-Sorbitol	SOR	18
Monosaccharides II	d	L-Rhamnose	RAM	16
Monosaccharides II	d	myo-Inositol	INS	35
Monosaccharides II	d	Xylitol	XYL	29
Cholic acid derivatives	e	CHAPS	CPS	84
Cholic acid derivatives	e	CHAPSO	1N7	35
Cholic acid derivatives	e	Glycocholic acid	GCH	5
Cholic acid derivatives	e	Taurocholic acid	TCH	10
Cryopolyols	f	1,1,1-Tris(hydroxymethyl)propane	n/a	0
Cryopolyols	f	1,2,4-Butanetriol	n/a	0
Cryopolyols	f	1,2,6-Hexanetriol	n/a	0
Cryopolyols	f	1,5-Pentanediol	n/a	0
Cryopolyols	f	meso-Erythritol	MRY	22
NDSBs	g	NDSB 195	NDS	10
NDSBs	g	NDSB 201	1PS	31
NDSBs	g	NDSB 221	KH2	4
NDSBs	g	NDSB 256	DMX	5
NDSBs	g	NDSB 211	n/a	0

Table S3. Details on the 7 commercially-available test proteins.

Protein	Origin	Buffer	Function	Organism	Production
α-Amylase	A9857	DW	Glycogen hydrolase	Aspergillus oryzae (fungus)	Aspergillus oryzae (fungus)
Avidin	A9275	DW	Biotin-binding protein	Chicken	Chicken egg
Catalase	C3155	DW (traces of Thymol)	Hydrogen peroxide hydrolase	Bovine	Bovine liver
Concanavalin A	L7647	DW	Lectin-binding protein	Canavalia ensiformis (Jack bean)	Canavalia ensiformis (Jack bean)
Insulin	I9278	Saline solution	Blood glucose hormone	Human	Saccharomyces cerevisiae
Lysozyme	L6876	DW	Glycoside hydrolase	Chicken	Chicken egg
Thaumatin	T7638	DW	Sweetener	Thaumatococcus daniellii (african plant)	Thaumatococcus daniellii (african plant)

Table S4. Crystallographic data summary table.

Sample	α-Amylase	Avidin	Catalase	Catalase	Catalase	Concanavalin A	Concanavalin A	Concanavalin A	Insulin	Insulin	Lysozyme	Thaumatin
Well	H11	H12	C4	F1	H4	B2	D8	E8	A12	A7	D4	G8
PDB code	7P4W	7P4Z	N/A	N/A	N/A	N/A	N/A	N/A	N/A	N/A	N/A	N/A
**Data processing**												
Space group	I222	C2	$P2_12_12_1$ (A)	$P2_12_12_1$ (B)	$P3_221$	$P2_12_12_1$	$P2_1$	I222	$I2_13$	R3	$P4_32_12$	$P4_12_12$
Unit cell parameters (Å)	65.7 / 140.4 / 154.2	114.4 / 43.3 / 62.3	86.4 / 140.0 / 231.9	68.5 / 171.9 / 192.1	140.6 / 140.6 / 101.8	66.5 / 116.2 / 122.7	59.9 / 64.2 / 126.1	63.9 / 88.0 / 89.4	77.7 / 77.7 / 77.7	79.2 / 79.2 / 37.3	78.5 / 78.5 / 37.0	57.9 / 57.9 / 150.8
Multiplicity	4.6 (4.3)	2.8 (2.7)	9.3 (8.7)	7.8 (3.3)	12.7 (6.9)	7.5 (1.0)	4.4 (3.2)	7.6 (3.2)	26.5 (20.7)	6.6 (4.0)	13.6 (1.1)	13.8 (1.4)
Completeness	99.8 (95.9)	99.9 (96.7)	100 (99.3)	92.9 (56.6)	98.5 (85.2)	97.9 (79.7)	89.8 (47.9)	96.4 (71.6)	100 (100)	99.7 (95)	85.1 (11.1)	83.4 (8.4)
CChalf	0.99 (0.28)	0.99 (0.97)	0.99 (0.29)	0.99 (0.31)	0.99 (0.34)	0.99 (0.43)	0.99 (0.33)	0.99 (0.42)	0.99 (0.35)	0.99 (0.17)	0.99 (0.37)	0.99 (0.90)
I/Sigma (I)	9.2 (0.4)	15.5 (2.7)	10.1 (0.6)	9.7 (0.7)	16.2 (0.4)	29.6 (0.7)	13.7 (0.2)	13.5 (0.1)	22.9 (0.4)	17.9 (0.3)	32.1 (0.4)	48.8 (1.2)
Rmerge	0.164 (2.2)	0.061 (0.23)	0.153 (2.30)	0.155 (1.34)	0.087 (2.27)	0.04 (0.225)	0.059 (1.93)	0.057 (2.79)	0.062 (3.52)	0.046 (1.75)	0.036 (0.53)	0.028 (0.194)
Resolution Range (Å)	51.9 – 2.28 (2.32 – 2.28)	58.9 – 2.2 (2.24 – 2.2)	73.5 – 2.24 (2.28 – 1.37)	64.6 – 1.50 (1.53 – 1.50)	57.9 – 1.61 (1.64 – 1.61)	61.4 – 1.37 (1.39 – 1.37)	59.8 – 1.57 (1.60 – 1.57)	52 – 1.53 (1.56 – 1.53)	55 – 1.53 (1.55– 1.53)	39.6 – 1.48 (1.50 – 1.48)	55.5 – 1.18 (1.2 – 1.18)	57.9 – 1.18 (1.2 – 1.18)
Unique Reflections	32915 (1549)	14917 (695)	135669 (6675)	335934 (10152)	147360 (6353)	136267 (5471)	119851 (3185)	36999 (1349)	11978 (602)	14545 (696)	32563 (211)	70760 (347)
Overall B factor ($Å^2$)	36.9	30.1	39.8	10.5	21.5	17.8	23.9	21.5	28.7	22.8	14.8	12.4
**Structure refinement**												
R factor (%)	22.8	24.7	N/A	N/A	N/A	N/A	N/A	N/A	N/A	N/A	N/A	N/A
R free (%)	28.5	29	N/A	N/A	N/A	N/A	N/A	N/A	N/A	N/A	N/A	N/A
Bond angle (degree)	1.14	1.21	N/A	N/A	N/A	N/A	N/A	N/A	N/A	N/A	N/A	N/A
Bond length (Å)	0.008	0.007	N/A	N/A	N/A	N/A	N/A	N/A	N/A	N/A	N/A	N/A
Molprobity score	1.58	1.56	N/A	N/A	N/A	N/A	N/A	N/A	N/A	N/A	N/A	N/A
